# Molecular Mechanisms of Curcumin on Diabetes-Induced Endothelial Dysfunctions: Txnip, ICAM-1, and NOX2 Expressions

**DOI:** 10.1155/2014/161346

**Published:** 2014-06-26

**Authors:** Natchaya Wongeakin, Parvapan Bhattarakosol, Suthiluk Patumraj

**Affiliations:** ^1^Center of Excellence for Microcirculation, Department of Physiology, Faculty of Medicine, Chulalongkorn University, Bangkok 10330, Thailand; ^2^Department of Microbiology, Faculty of Medicine, Chulalongkorn University, Bangkok 10330, Thailand

## Abstract

We aim to investigate the effects of curcumin on preventing diabetes-induced vascular inflammation in association with its actions on Txnip, ICAM-1, and NOX2 enzyme expressions. Male Wistar rats were divided into four groups: control (CON), diabetic (DM; streptozotocin (STZ), i.v. 55 mg/kg BW), control-treated with curcumin (CONCUR; 300 mg/kg BW), and diabetes treated with curcumin (DMCUR; 300 mg/kg BW). 12th week after STZ injection, iris blood perfusion, leukocyte adhesion, Txnip, p47phox, and malondialdehyde (MDA) levels were determined by using laser Doppler, intravital fluorescent confocal microscopy, Western Blot analysis, and TBAR assay, respectively. The iris blood perfusion of DM and DMCUR was decreased significantly compared to CON and CONCUR (*P* < 0.001). Plasma glucose and HbA1c of DM and DMCUR were increased significantly compared to CON and CONCUR (*P* < 0.001). Leukocyte adhesion, ICAM-1, p47phox expression, and MDA levels in DM were increased significantly compared to CON, CONCUR, and DMCUR (*P* < 0.05). Txnip expression in DM and DMCUR was significantly higher than CON and CONCUR (*P* < 0.05). From Pearson's analysis, the correlation between the plasma MDA level and the endothelial functions was significant. It suggested that curcumin could ameliorate diabetic vascular inflammation by decreasing ROS overproduction, reducing leukocyte-endothelium interaction, and inhibiting ICAM-1 and NOX2 expression.

## 1. Introduction

Diabetes-induced hyperglycemia can disturb the vascular homeostasis that is characterized by decreased vascular blood perfusion, increased vascular permeability, and enhanced vascular inflammation, leading to diabetic vascular complications. Several studies [1–4] reported that hyperglycemia-induced reactive oxygen species (ROS) overproduction are continuously produced during metabolic processes through the pathways including NADPH oxidase, xanthine oxidase, and mitochondria respiratory chain. ROS overproduction above the physiological level can overcome the functions of cellular antioxidant system leading to oxidative stress.

Hyperglycemia could induce high-level thioredoxin-interacting protein (Txnip) expression [5, 6] by stimulating carbohydrate response element binding protein and forkhead box01 transcription factor [7, 8]. Txnip is an endogenous inhibitor of thioredoxin (TRX) system which is one of the important cellular antioxidant systems. The Txnip-TRX interaction can produce multiple processes consequently including increasing intracellular ROS [9–11], activating stress signaling pathway of apoptosis signal-regulating kinase 1 (ASK-1), increasing intercellular adhesion molecule 1 (ICAM-1) expression, and finally resulting in endothelial dysfunction [10, 12–14].

Through the other pathway, hyperglycemia-induced ROS can stimulate NF-*κ*B activation which then causes the increase in vascular adhesion molecule expression. Therefore, it implies that both pathways of Txnip-TRX interaction and NF-*κ*B activation refer to promoting leukocyte-endothelium interaction and play a central role in diabetic vascular inflammation [12, 15, 16]. Moreover, the severity of diabetic vascular inflammation reproduced after the plugged leukocyte in microcirculation via the process of phagocytic NADPH oxidase (NOX2 enzyme) mediated superoxide radical formation [17–19].

Recently, the use of natural plant products has gained more attention among scientists in order to prevent diabetic vascular complications. Curcumin is one of the polyphenol compounds which exhibit antioxidant, anti-inflammatory, antitumorigenic, and antimicrobial properties. With its antioxidant activity, curcumin has direct effect to scavenge free radicals by redox activity [20–22] and indirect effect to enhance cellular antioxidant system by activating Nrf2 transcription factor, a cytoprotective gene [23–26]. With its anti-inflammatory activity, curcumin can attenuate proinflammatory cytokine levels such as TNF-*α*, IL-1*β*, and IL-6 by interfering with the activation of NF-*κ*B, c-Jun, and JNK [27–30]. Several studies [31, 32] found that curcumin can reduce adhesion molecule by inhibiting proinflammatory cytokine secretion in in vitro model. Also, our previous study found that curcumin can attenuate leukocyte-endothelium interaction and ROS overproduction represented by MDA levels in STZ-induced diabetic rat model [33, 34].

Based on these reviews, we hypothesized that the curcumin supplementation which is able to reduce leukocytes adhesion may be achieved by its antioxidative effects on Txnip and ICAM-1 expression and also the inhibition of phagocytic/leukocyte action of NOX2-generated O_2_^−^. Therefore, the present study aims to investigate the mechanism of curcumin on diabetes-induced vascular complications related to these molecular biomarkers including Txnip, ICAM-1, and NOX2 expressions using STZ-induced diabetic rat model.

## 2. Materials and Methods

### 2.1. Treatment of Animals

Male Wistar rats (220–250 g) were obtained from National Laboratory Animal Center, Salaya Campus, Mahidol University, Thailand. All rats were randomly divided into diabetic and nondiabetic groups. The diabetic group was induced by streptozotocin (STZ, Sigma Co., USA) at dose 55 mg/kg BW, i.v. [33, 34]. The diabetic condition was defined as a glucose concentration more than 200 mg/dL [35] and verified at 48 hours after STZ injection. The diabetic rats were divided into two groups: diabetes treated with corn oil (DM; *n* = 6) and diabetes treated with curcumin (DMCUR, *n* = 6, 300 mg/kg BW, Cayman, USA). In the control groups, they will receive the same volume of citrate buffer. They were divided into two groups: control treated with corn oil (CON, *n* = 6) and control treated with curcumin (CONCUR, *n* = 6). The dairy gavage feeding of curcumin dissolved in corn oil was started on 10th day after the STZ injection.

### 2.2. Experiment of Animals

On the 12th week after STZ injection, the rat was anesthetized with pentobarbital sodium (60 mg/kg BW i.p.), the rats were kept warm at 37°C using warming pad, and a tracheotomy was performed. A jugular vein and carotid artery were cannulated with polyethylene tube for injection of fluorescence tracers and for recording of systolic and diastolic blood pressure, respectively. The blood pressure was measured by using Statham pressure transducer connected to the Polygraph system (Nihon Koden, Japan). Mean arterial blood pressure was calculated from diastolic pressure + 1/3(systolic-diastolic).

### 2.3. Iris Blood Perfusion Measurement

The right iris blood perfusion was measured using the Laser Doppler Blood Perfusion Monitoring (Perimed AB, Sweden) with the optic needle probe (0.1 millimeter). The needle probe was fixed perpendicularly about 1 mm above iris. Eight different measurement points of iris around pupil were performed at each time and the mean of iris blood perfusion was determined for each rat.

### 2.4. Evaluation of Leukocyte Adhesion

The iris blood vessel was used to observe instead of retina because the microcirculation in the iris is quite properly to get the good image of leukocyte-endothelial cell interaction and to assess blood perfusion. Besides, the diabetic iridopathy is commonly used clinically to indicate the progression of the diabetic microvascular complications, particularly, in association with advanced proliferative diabetic retinopathy by using fluorescein angiography of the iris [36].

During the experiment, the real time image of iris blood vessel was recorded by an epi-illumination fluorescence video microscopy system (Optiphot 2, Nikon, Japan) equipped with a 100 W mercury lamp, CCD camera (Hamamatsu C2400, Japan), a video recorder (VC-S5, Sharp, Japan) with a video timer (VTG-33, For-A, Japan), and 20x objective lens (CF Plan Fluor, Nikon, Japan).

Leukocyte adhesion in postcapillary venules of iris was determined by rhodamine-6G (R6G) which can label mitochondria especially in leukocyte (Sigma, USA) at 0.15 mg/kg BW following the method described by Jariyapongskul et al. [37]. The emission wavelength of R6G lies between 530 and 540 nm. rhodamine-6G was injected intravenously for visualizing the leukocyte adhesion. Five observation views were chosen and recorded to further determine leukocyte adhesion [38]. The leukocytes which remained stationary to postcapillary venules for 30 seconds were considered to be leukocyte adhesion [33, 39]. The number of leukocyte adhesions in each view was manually counted and reported by the mean number of cells per field of view.

### 2.5. Biochemical Parameters

The parameters for metabolic changes were blood glucose and HbA1c. All these were determined at the end of the experiments by collected blood sample from abdominal aorta under anesthesia. Blood glucose and HbA1c were measured using enzymatic method and turbidimetric immunoinhibition method, respectively (Bangkok RIA Laboratory Co., Bangkok, Thailand).

In this study fundus malondialdehyde level (MDA), product of lipid peroxidation, was used as indicator of oxygen free radicals in fundus. Thiobarbituric acid (TBA) assay is a commonly used method to determine malondialdehyde. This method is based on the reaction of TBA and MDA. The lipid material is simply heated with TBA under acidic condition, and the formation of a pink color is measured at or close to 532 nm. The pink color is formed by reaction of one molecule of MDA with two molecules of TBA [37].

### 2.6. Protein Assay

After collecting blood, the eyeball was collected immediately by enucleating from the orbital cavity. Lens and eye fluid were excised from the eye ball; the eyeball without lens and fluids is called fundus. The fundus was washed in phosphate buffer (0.1 M, pH 7.4). Then, the fundus was chopped with fine scissors and was directly in lysis buffer containing 1X RIPA buffer (250 *μ*L of lysis solution per 25 mg of tissue, Cell Signaling, Beverly, MA), 1X phosphatase inhibitor cocktails (1 : 100, Sigma Co., USA), and 1X protease inhibitor (1 : 100, Sigma Co., USA) for 30 minutes [40]. After that, the chopped fundus was sonicated for 3 times for 10 seconds each and was centrifuged at 15,000 g for 10 minutes to spin down cellular debris. The supernatant was collected as the whole cell lysate to determine protein concentration [41]. After protein quantification, the samples were used to determine MDA level by method of Ohkawa et al. [42] and Txnip, ICAM-1, and p47phox expressions by Western blot analysis [6, 7, 21, 24].

### 2.7. Western Blot Analysis

The protein expressions of Txnip, ICAM-1, and p47phox were performed with 80 *μ*g of fundus protein extraction per lane by 10% SDS-PAGE gel (Bio-Rad Laboratories), transferred to polyvinylidene difluoride (PVDF) membrane, and blocked with blocking solution (5% BSA (Sigma Co., USA) in TTBS). After blocking, the PVDF membrane was incubated with either anti-mouse Txnip antibody (1 *μ*g/mL, MBL, IL), anti-mouse ICAM-1 antibody (1 : 500, BD Biosciences, CA), anti-mouse p47phox antibody (1 : 200, Santa Cruz, CA), or anti-mouse *β*-actin antibody (1 : 200, Santa Cruz, CA) and followed by horseradish-peroxidase conjugated goat anti-mouse secondary antibody at 1 : 2000 dilution (Santa Cruz, CA). The PVDF membrane was incubated with chemiluminescence substrate solution (GE, USA) and the bands were detected using ChemiDoc system (Bio-Rad Laboratories) with Quantity One program version 4.6.9. Then, densitometry was performed using Image J analysis software (NIH) and the results of Txnip, ICAM-1, and p47phox expressions were quantified as a ratio to *β*-actin expression.

### 2.8. Statistical Analysis

All data were presented as means ± SEM (standard error of mean). For comparison among groups of rats, two-way analysis of variance (two-way ANOVA) was used and followed by unpaired *t*-test. *P* < 0.05 was considered statistically significant. The association of each parameter was analyzed by using two-tailed Pearson's correlation analysis with *P* < 0.05 being considered statistically significant. The data was analyzed using the SPSS program (version 16.0) for windows.

## 3. Results

### 3.1. The Effects of Curcumin on Physiological Characteristics

As shown in Table 1, the body weights (BW) of 12-week DM and DMCUR rats were significantly decreased (35.93% and 39.15%) as compared to CON and CONCUR groups (*P* < 0.001). Mean arterial blood pressure (MAP) of 12-week DM and DMCUR groups was not significantly different as compared to CON and CONCUR groups (Table 1).

### 3.2. The Effects of Curcumin on Biochemical Parameters

Blood glucose levels (BG) of DM group and DMCUR groups were significantly elevated as compared to CON group and CONCUR group (*P* < 0.001).

Plasma glycosylated hemoglobin levels of DM group and DMCUR group were significantly elevated as compared to CON group and CONCUR group (*P* < 0.001) (Table 2). The results seem to indicate that the curcumin (300 mg/kg BW) could not inhibit high BG significantly.

### 3.3. The Effects of Curcumin on Hemodynamic Changes

**Figure 1:**
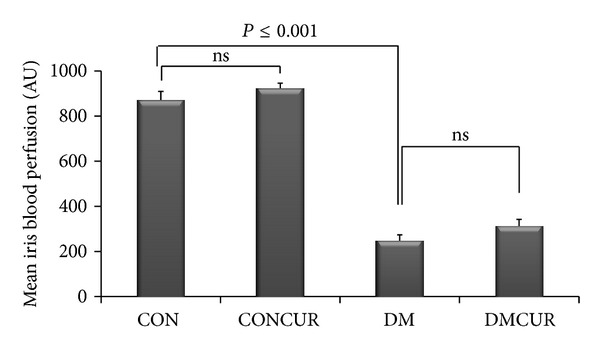
The effects of curcumin on mean regional iris blood perfusion in each group. Control = CON, control treated with curcumin = CONCUR; curcumin 300 mg/kg BW, started at 10 days after STZ injection, diabetic = DM; streptozotocin (STZ), i.v. 55 mg/kg BW, diabetes treated with curcumin = DMCUR; curcumin 300 mg/kg BW. Data are means ± SEM (*n* = 6 for each group).

By using laser Doppler flowmetry, the regional iris blood perfusion of each rat was evaluated from eight different points measured around the pupil as described previously. Mean regional iris blood perfusion in each group was summarized in Figure 1. Mean iris blood perfusion (IBP) of DM group was significantly reduced as compared to CON and CONCUR groups (*P* < 0.001). IBP in DMCUR group showed a trend to increase but not significantly different when compared to DM group.

### 3.4. The Effects of Curcumin on Leukocyte-Endothelium Interaction

The number of leukocytes was counted as adherent one that remained stationary for equal to or longer than 30 seconds. The leukocyte adhesion was counted per field of view totally of postcapillary venule (diameter 20–30 *μ*m) as described previously.

**Figure 2:**
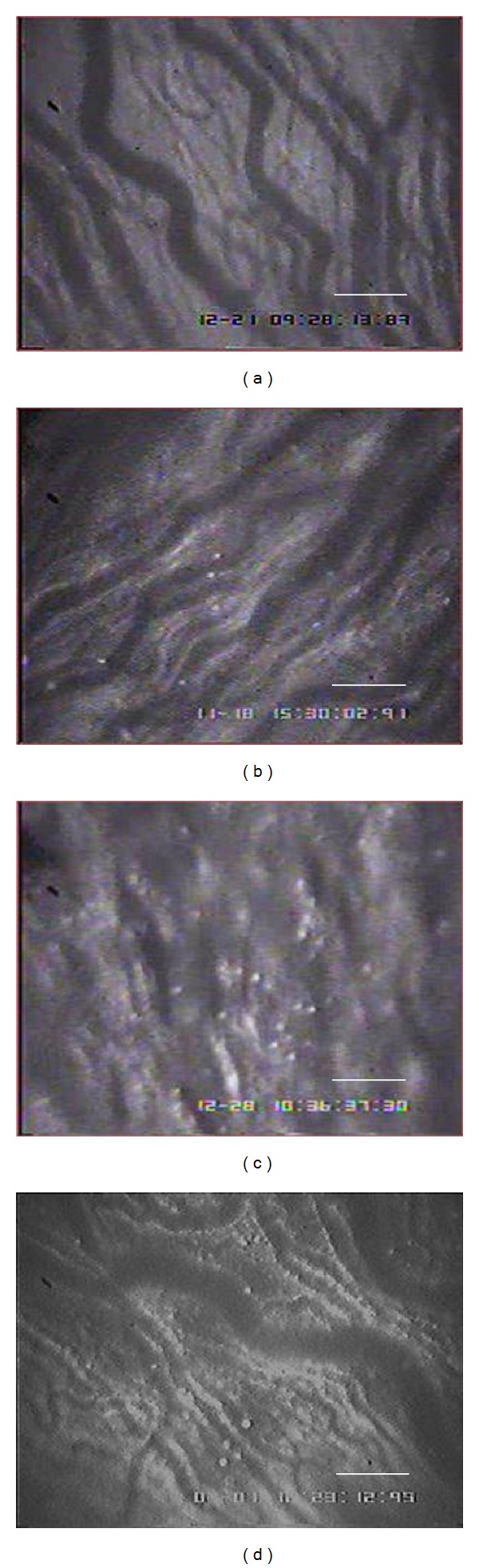
Intravital microscopic demonstration of leukocyte adhesion in the postcapillary venule of iris in CON group (a), CONCUR group (b), DM group (c), and DMCUR group (d). White dots represent leukocyte stained by rhodamine-6G i.v. injection. (Scale bar represents 100 *μ*m.)

**Figure 3:**
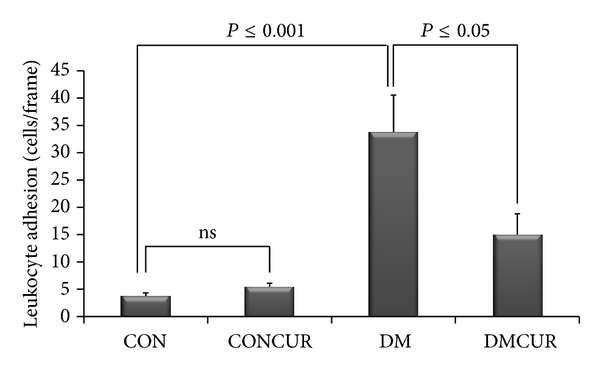
The effects of curcumin on leukocyte-endothelium interaction. Data are means ± SEM (*n* = 5 for each group). Control = CON, control treated with curcumin = CONCUR; curcumin 300 mg/kg BW, started at 10 days after STZ injection, diabetic = DM; streptozotocin (STZ), i.v. 55 mg/kg BW, diabetes treated with curcumin = DMCUR; curcumin 300 mg/kg BW.

In the present video microscopic visualization showed clear image of leukocyte adhering to the endothelium of postcapillary venule in the different monitored views of iris of each rat (Figure 2). Five different monitored views of leukocyte adhesion were manually counted and the mean of leukocyte adhesion per field of views were summarized in the graph as shown in Figure 3.

The number of leukocyte adhesions was significantly increased in DM group as compared to CON group and CONCUR group (*P* < 0.001). Interestingly, the number of leukocyte adhesions of DMCUR group was significantly reduced as compared to DM group (*P* < 0.05) (Figure 3).

### 3.5. The Effects of Curcumin on Free Radicals by Products

**Figure 4:**
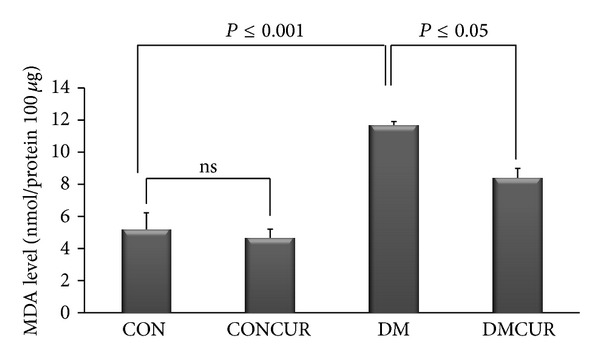
The effects of curcumin on fundus MDA level. Control = CON, control treated with curcumin = CONCUR; curcumin 300 mg/kg BW, started at 10 days after STZ injection, diabetic = DM; streptozotocin (STZ), i.v. 55 mg/kg BW, diabetes treated with curcumin = DMCUR; curcumin 300 mg/kg BW. Data are means ± SEM (*n* = 5 for each group).

In this study fundus malondialdehyde level (MDA), product of lipid peroxidation, was used as indicator of oxygen free radicals in fundus. MDA level was significantly elevated in DM group as compared to CON group and CONCUR group (*P* < 0.001). Interestingly, MDA level of DMCUR group was significantly reduced as compared to DM group (*P* < 0.05) (Figure 4).

### 3.6. The Effects of Curcumin on Txnip Expression

**Figure 5:**
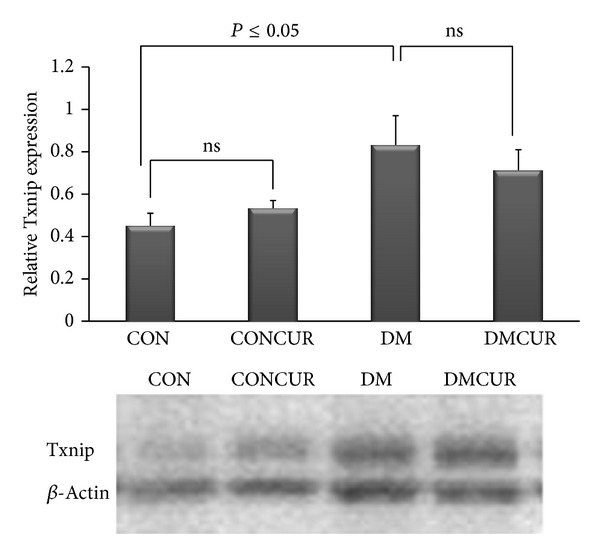
The effect of curcumin on Txnip expression. Control = CON, control treated with curcumin = CONCUR; curcumin 300 mg/kg BW, started at 10 days after STZ injection, diabetic = DM; streptozotocin (STZ), i.v. 55 mg/kg BW, diabetes treated with curcumin = DMCUR; curcumin 300 mg/kg BW. Data are means ± SEM (CON and DMCUR groups; *n* = 5, CONCUR and DM groups; *n* = 6).

The level of Txnip was significantly elevated in DM group and DMCUR group as compared to CON group (*P* < 0.05). However, the results showed that curcumin could not reduce Txnip expression significantly when compared to the DM group. (*P* < 0.05) (Figure 5).

### 3.7. The Effects of Curcumin on ICAM-1 Expression

**Figure 6:**
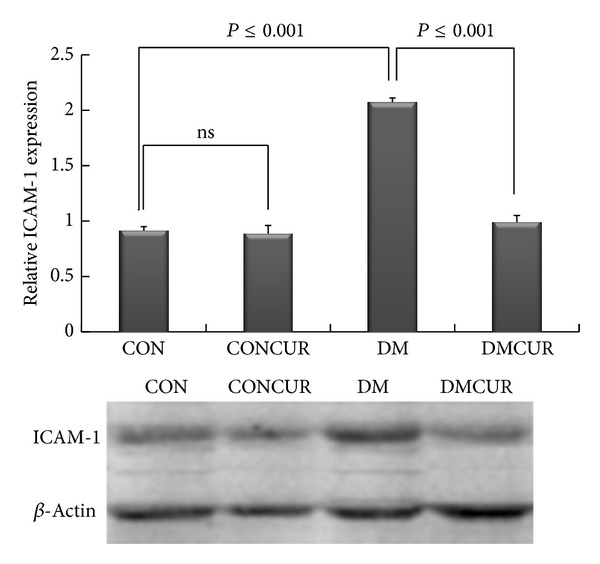
The effect of curcumin on ICAM-1 expression. Control = CON, control treated with curcumin = CONCUR; curcumin 300 mg/kg BW, started at 10 days after STZ injection, diabetic = DM; streptozotocin (STZ), i.v. 55 mg/kg BW, diabetes treated with curcumin = DMCUR; curcumin 300 mg/kg BW. Data are means ± SEM (*n* = 4).

The level of ICAM-1 was significantly elevated in DM group as compared to CON group (*P* < 0.05). The level of ICAM-1 was significantly decreased in DMCUR compared to DM group (*P* < 0.05) (Figure 6).

### 3.8. The Effects of Curcumin on p47phox Expression

**Figure 7:**
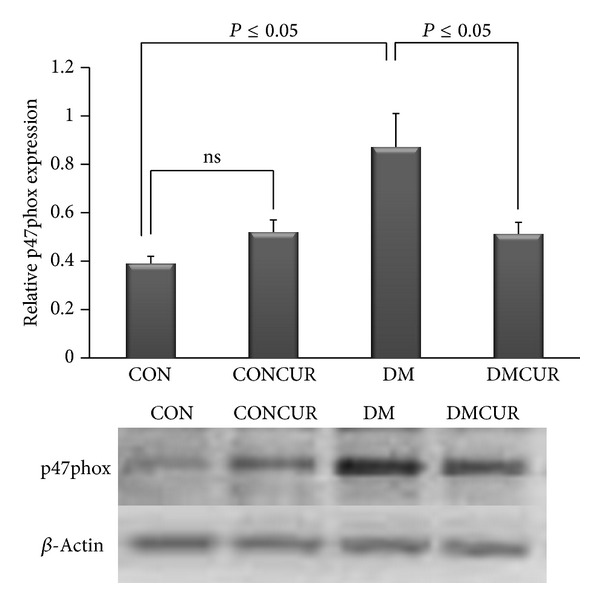
The effect of curcumin on p47phox expression. Control = CON, control treated with curcumin = CONCUR; curcumin 300 mg/kg BW, started at 10 days after STZ injection, diabetic = DM; streptozotocin (STZ), i.v. 55 mg/kg BW, diabetes treated with curcumin = DMCUR; curcumin 300 mg/kg BW. Data are means ± SEM (CON, CONCUR, and DM groups; *n* = 5, and DMCUR group; *n* = 4).

The level of p47phox was significantly elevated in DM group as compared to CON group and CONCUR group (*P* < 0.05). Interestingly, the level of p47phox of DMCUR group was significantly reduced as compared to DM group, approximately 1.71-fold (*P* < 0.05) (Figure 7).

**Figure 8:**
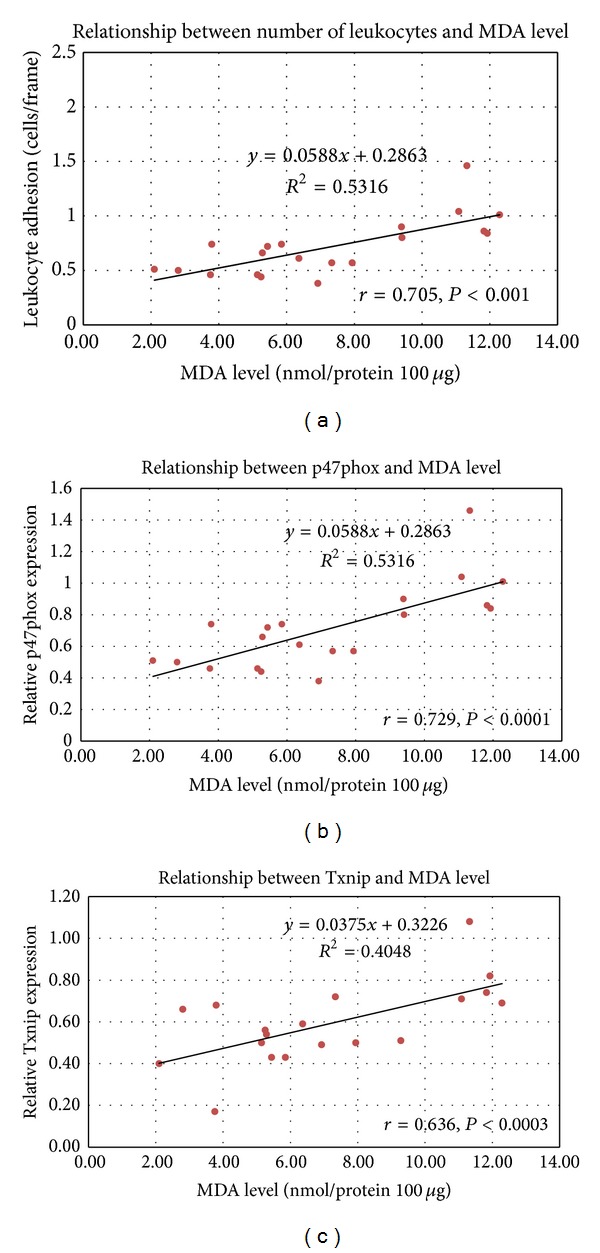
The association between the biochemical and functional data parameters was analyzed by using two-tailed Pearson's correlation analysis with *P* < 0.05 being considered statistically significant.

From Figure 8, Pearson's correlation between the leukocyte adhesion and MDA level, p47phox and MDA level, and Txnip andMDA level was obtained together with Pearson's correlation and significance: *r* = 0.705  (*P* = 0.001), *r* = 0.729  (*P* = 0.0001), and *r* = 0.636  (*P* = 0.0003),* respectively*.

## 4. Discussion

### 4.1. The Mechanisms of Curcumin on Leukocyte-Endothelium Interaction:* Roles of Txnip, ICAM-1, and p47phox Expressions*

This experiment demonstrated that curcumin supplementation can attenuate leukocyte-endothelium interaction in association with the decreased ROS overproduction. In addition, curcumin can also suppress NOX2 enzyme expression in vascular inflammatory process in diabetic rats. However, the curcumin supplementation (300 mg/kg BW) could not show the significant inhibition of the hyperglycemia-enhanced Txnip expression when compared to the DM group.

From the previous studies, hyperglycemia was reported as an important factor to induce Txnip expression to increase. Moreover, the hyperglycemia-enhanced Txnip expression has been noted to play a critical role in progression of diabetic vascular complications as well [6, 16, 43, 44]. As shown in our data, Txnip expression in DM group was significantly increased up to 84.44% when compared to the CON group (*P* = 0.045). The results showed that curcumin could not reduce Txnip expression as that it conforms to the results shown no hypoglycemic effects for curcumin in DMCUR. Since curcumin could not inhibit high BG, therefore, hyperglycemia-enhanced Txnip expression was still existent. Actually, the hypoglycemic effect of curcumin is still controversy. Some studies have reported that curcumin supplementation can ameliorate hyperglycemia in both type 1 [33, 45, 46] and type 2 diabetic models [47] but some did not support this [21, 48, 49].

It is noted that Txnip expression in DMCUR group has tended to slightly reduce down to 14.46% compared to DM group (Figure 5). It may explain that at dose of 300 mg/kg BW curcumin used in this study may not be able to decrease BG and HbA1c; therefore, it was not enough to prevent the alteration of Txnip expression in response to hyperglycemia.

In addition to hyperglycemia-regulated Txnip overexpression, hyperglycemia also can activate many sources to generate ROS such as mitochondria electron transport chain, NADPH oxidase, xanthine oxidase, endothelial NO synthase, and cytochrome p450 [1–4].

The experiments confirmed that hyperglycemia promotes ROS overproduction resulting oxidative stress to damage macromolecules such as lipid which is in apparently the increasing of fundus MDA level in DM group. However, the fundus MDA level in DMCUR group was significantly decreased down to 28% as compared to DM group (*P* = 0.005). The results of MDA level implied the potential of curcumin as an effective antioxidant.

Both Txnip expression and ROS overproduction could impair endothelial function that causes vascular homeostasis alteration including the abnormal vascular blood flow resistance. In this experiment, blood perfusion in iris was chosen to represent one of the alterations of vascular homeostasis. The results showed that iris blood perfusion (IBP) in DM and DMCUR groups was significantly decreased as compared to CON group, though IBP in DMCUR had tended to increase up to 4.78% as compared to DM group. However, these results did not show the significant difference similar to the previous studies [33, 34]. There are several studies that reported that curcumin could increase cerebral blood flow and improve endothelium-dependent relaxation in mesentery of diabetes rats [50–52]. Therefore, the antioxidant potential of curcumin should be different depending on doses and also the pathological condition of those organs including the severity of cellular antioxidant system [52, 53].

Furthermore, our results have shown that the low dose of curcumin supplementation could significantly decrease ICAM-1 expression down to 52.17% and the number of leukocyte-endothelium interactions was down to 55.68% as compared to the CON group (Figures 3 and 6). Several studies indicated that the enhancing of adhesion molecule expression such as ICAM-1 and VCAM-1 is associated with Txnip-activated ASK-1 stress signaling pathway and ROS-suppressed nitric oxide bioavailability resulting in vascular inflammation [12–14, 54]. Therefore our results may imply that the anti-inflammatory effect of curcumin contributed to prevention of leukocyte adhesion partly by reducing ICAM-1 expression. The effect of curcumin on proinflammatory cytokines such as IL-1 and IL-6 via blockade of Akt, p38MAPK, and NF-*κ*B pathways might be the major processes that explained our observation [28, 29, 33, 55, 56].

From Figure 7, the results of p47phox expressions indicated that the expression of NOX2 enzyme was significantly increased in DM group when compared to the CON group (*P* = 0.05). The increased p47phox, one of the NOX2 multienzyme complexes in diabetes, was demonstrated by other reports as well [19, 57–59]. It is believed that this phagocytic NADPH oxidase (NOX2 enzyme) is expressed by both phagocytic and vascular endothelial cells. Through this phagocyte NADPH oxidase, ROS could be produced at high levels after leukocyte and endothelial cell activations and then it will cause more diabetic vascular injury [18, 19, 60]. Thus, it seems likely that both phagocyte- and endothelial cell-derived ROS are critically involved in the diabetes-induced vascular pathologic complications.

Our findings indicated that the p47phox expression in DMCUR group was significantly decreased down to 41.38% as compared to DM group. At this point, it may imply that when curcumin could decrease ICAM-1 and NOX2 enzyme expressions, it will inhibit the leukocyte adhesion and prevent vascular inflammation in consequence. Moreover the data from our study also demonstrated the correlation between the plasma MDA level and the endothelial functions significantly (Figure 8). By this significant correlation, it further confirmed the possible protective effects of curcumin against diabetes-induced vascular pathologic complications via the antioxidant mechanism.

## 5. Conclusions

In summary, our experiments demonstrated that low dose curcumin supplementation (300 mg/kg BW) can ameliorate diabetic vascular inflammation through the decrease in ROS overproduction, reducing leukocyte-endothelium interaction associated with decreased ICAM-1 and NOX2 enzyme expressions in diabetic rat model. Therefore the findings of this study should be a good evidence to suggest that the low dose of curcumin might offer a novel therapeutic strategy for treatment of the diabetic retinopathy associated with vascular inflammation in the future.

## Figures and Tables

**Table 1:** Values are means ± SEM.

Group	BW (g)	MAP (mmHg)
12-week CON (*n* = 6)	424.00 ± 28.28	124.77 ± 9.60
12-week CONCUR (*n* = 6)	480.67 ± 17.26	132.58 ± 5.50
12-week DM (*n* = 6)	271.67 ± 13.22**	113.56 ± 6.98
12-week DMCUR (*n* = 6)	258.00 ± 17.64**	112.80 ± 4.99

**P < 0.001 versus CON.

**Table 2:** Values are means ± SEM.

Group	BG (mg/dL)	Hemoglobin A1c (%)
12-week CON (*n* = 6)	172.83 ± 11.08	4.00 ± 0.06
12-week CONCUR (*n* = 6)	192.50 ± 13.05	4.08 ± 0.05
12-week DM (*n* = 6)	410.33 ± 16.77**	9.47±0.47∗∗
12-week DMCUR (*n* = 6)	390.33 ± 11.59**	9.30 ± 0.21**

**P < 0.001 versus CON.

## Abbreviations

DM: Diabetes mellitus
IBP: Iris blood perfusion
ICAM-1: Intercellular adhesion molecule 1
MDA: Malondialdehyde levels
MAP: Mean arterial blood pressure
NOX2: NADPH oxidase enzyme
R6G: Rhodamine-6G
ROI: Region of interest
ROS: Reactive oxygen species
STZ: Streptozotocin
O_2_^−^: Superoxide
Txnip: Thioredoxin-interacting protein.
